# The world congress on insulin resistance, diabetes, and cardiovascular disease (WCIRDC)

**DOI:** 10.1111/1753-0407.13260

**Published:** 2022-02-22

**Authors:** Zachary Bloomgarden

## 1 THE BETA CELL IN DIABETES

The meeting began with a discussion addressing the underrecognized importance of the beta cell in type 2 diabetes (T2D). Mohammad Abdul‐Ghani, San Antonio, TX, presented evidence of beta cell failure in T2D, discussing the Efficacy and Durability of Initial Combination Therapy for Type 2 Diabetes (EDICT) study of 323 persons newly found to have T2D randomized to conventional sequential treatment with metformin, followed by a sulfonylurea, and then by insulin, or to initial treatment with a combination of metformin, pioglitazone, and exenatide. Over 72 months, the latter intervention was effective even with initial HbA1c > 9%, appearing to be mediated by sustained improvement in beta cell function as well as in insulin sensitivity.^1^ In the study, approximately one quarter of those randomized to metformin alone had sustained stable glycemic control with metformin, with Abdul‐Ghani showing evidence that these were the patients with higher baseline levels of beta‐cell function, as measured using the ratio of C‐peptide levels before to that 120 min after glucose loading, whereas baseline HbA1c was not predictive of response to metforminmonotherapy.^1^, ^2^ The similarly structured Qatar study enrolled patients with more longstanding T2D on metformin and sulfonylurea randomized to the addition of pioglitazone plus a glucagon‐like peptide‐1 receptor agonist (GLP‐1RA) vs addition of insulin, similarly showing sustained improvement with the former approach.^3^ Abdul‐Ghani reviewed the evidence of improvement in beta cell function of the GLP‐1RA liraglutide,^4^ but also showed improved insulin secretion with pioglitazone,^5^ over time well‐exceeding the effect of sulfonylurea.^6^

Jack L. Leahy, Burlington, VT, discussed evidence that elevated levels of glucose and lipids play roles in beta cell dysfunction in T2D, proposing a self‐perpetuating cycle in which the inability of the beta cell to fully compensate for the metabolic stresses beginning at the onset of glucose intolerance is followed by progressive decline in beta cell mass and function resulting in further worsening of hyperglycemia, perhaps a direct effect of hyperglycemia, but potentially attributable to “beta‐cell exhaustion,” perhaps caused by endoplasmic reticular or oxidative stress, or perhaps a consequence of elevations in free fatty acids (FFA). However, Leahy pointed out that lipid infusion to elevate FFA levels reduces insulin secretion only in persons with positive family history of T2D.^7^ Additional potential mechanisms of the detrimental effect of hyperglycemia include amyloid or inflammatory infiltration of islets and epigenic changes. Importantly, studies carried out more than 4 decades ago demonstrated improved insulin secretion after a period of glycemic normalization,^8^, ^9^ so that there may be a variable initial period of potentially reversible beta‐cell dysfunction, after which beta‐cell mass progressively declines.^10^ The initial reversible period can, Leahy suggested, be one of beta‐cell dedifferentiation, as suggested by downregulation of β‐cell insulin, glucokinase, pancreatic and duodenal homeobox 1PDX‐1, and peroxisome proliferator‐activated receptor alpha (PPARα) gene expression, whereas hexokinase, lactate dehydrogenase, and PPARγ gene expression are upregulated in a 90% pancreatectomy model^11^ and with high fat feeding.^12^

Rohit N. Kulkarni, Boston, MA, reviewed modifications in mRNA as mediators of the inability of the beta cell to compensate in T2D for insulin resistance by appropriately increasing insulin secretion. The main RNA modification is the formation of N6‐methyladenosine (m^6^A), a reversible process that has an important role in neuronal development^13^ and in beta cell survival, growth and secretory function.^14^ Beta cell insulin signaling is also affected by changes in methylation both of insulin‐like growth factor and of insulin receptors. Steven Kahn, Seattle, WA, reviewed evidence of genetic polymorphisms involved in beta cell failure, contributing to the decrease in insulin secretory function of T2D development,^15^ noting that different genes involved in various aspects of beta cell function can be characterized, and play roles in differing type 2 diabetes presentations,^16^ such as those of youth versus adult T2D.^17^

Ralph DeFronzo, San Antonio, TX, discussed potential roles of different antidiabetic agents in addressing beta cell failure. He commented that T2D is based on the combination of tissue insulin resistance with impairment in insulin secretion, with more than half of beta‐cell function lost before T2D onset, leading him to note that impaired glucose tolerance is “really advanced diabetes as far as I’m concerned,” as shown in the San Antonio study of pre‐T2D.^18^ He pointed out that many treatment approaches, such as sulfonylurea, metformin, insulin and the dipeptidyl peptidase 4 inhibitors have little effect on beta cell function, and noted that metformin should not be considered an insulin sensitizer, contrasting this with the combined benefit of thiazolidinediones in improving both insulin sensitivity and insulin secretion,^19^ with evidence that the GLP‐1 receptor activators also have beneficial effects on beta‐cell function^20^ and that the sodium glucose transporter 2 inhibitors improve insulin action.^21^

Richard Pratley, Orlando, FL, discussed the evidence of latent autoimmune diabetes of adults (LADA) having similarities to both type 1 diabetes (T1D) and T2D. Approximately half of type 1 diabetes occurs in adults, whereas 5%–10% of persons with T2D have positive glutamic acid decarboxylase (GAD) antibodies, but genetic association studies show that transcription factor 7‐like 2 polymorphisms are associated with LADA, and approximately 40% of persons with LADA are overweight or obese, making it appear that there are similarities to T2D as well. Peter Reaven, Phoenix, AZ, brought together observations pertaining to relationships between C‐peptide levels and cardiovascular disease (CVD) outcomes. Low C‐peptide appears to be a measure of decreased endogenous insulin secretion in T2D as well as in T1D,^22^ and long‐term follow‐up of patients with T2D shows low C‐peptide to be associated with microvascular complications, although not with mortality.^23^ Reaven reviewed studies with C‐peptide measurements on participants in the Veteran’s Administration Diabetes Trial, showing that those in the lowest quartile had longer diabetes duration, and more use of insulin, whereas the highest quartile had lower high‐density lipoprotein (HDL) cholesterol and higher triglyceride (TG) levels, suggesting greater degrees of insulin resistance. Those with the lowest C‐peptide had higher levels of HbA1c, greater glucose variability, and greater likelihood of developing hypoglycemia. Similar findings were reported in the Action to Control Cardiovascular Risk in Diabetes (ACCORD) trial of the relationship between C‐peptide and risk of severe hypoglycemia.^24^ CVD events were more common both in the lowest and highest C‐peptide quartile, an association not explained by treatment assignment, HbA1c, hypoglycemia, or glucose variability. Both the C‐peptide and LADA studies suggest that there is high heterogeneity of T2D, with implications that better patient characterization may be useful in treatment decisions, perhaps using C‐peptide measurement in characterizing different T2D phenotypes. Additional measurement of insulin resistance and insulin secretion, as well as obesity and GAD antibody measurements, may further optimize T2D treatment approaches.^25^

## 2 INSULIN RESISTANCE: TISSUE‐SPECIFIC CONCEPTS

Ronald Krauss, Berkeley, CA, discussed the association between the metabolic syndrome and atherogenic dyslipidemia, with high TG levels, TG‐rich lipoproteins and partially catabolized remnants (very low‐density lipoprotein [VLDL] and intermediate‐density lipoprotein [IDL]), Low levels of HDL cholesterol, mainly due to reduced large HDL particles, and increased numbers of small dense cholesterol‐depleted LDL (sdLDL) particles, without increase in LDL cholesterol but with increased levels of apolipoprotein B (apoB), the structural protein of LDL, IDL, and VLDL, which can be considered a measure of total particle number. Individuals with LDL phenotype B, defined by predominance of sdLDL particles, show a cut point at a TG level of 95 mg/dL,^26^ considerably lower than the TG typically thought of as marking insulin resistance. Small LDL particles are formed from metabolism of large VLDL by lipoprotein lipase (LPL) to IDL remnants, which in turn are metabolized by LPL and hepatic lipase to progressively smaller LDL particles,^27^ which have 60% longer plasma LDL residence time than the larger LDL particles formed from small VLDL under the action of LPL.^28^ A meta‐analysis of more than 250 000 persons from 29 studies showed that fasting TG levels are associated with increased coronary heart disease (CHD) risk.^29^ Krauss commented, “it’s not the TG itself, it’s the remnants,” reviewing a combined observational and Mendelian randomization study suggesting a stronger relationship of remnants than of LDL cholesterol to CHD risk.^30^ A more recent study showed significant association of the combination of high LDL with high levels of remnant cholesterol with risk,^31^ underscoring the limited risk associated with larger LDL size,^32^, ^33^ whereas there is a dose‐response relationship between either sdLDL cholesterol or sdLDL particle number and CVD risk.^34^, ^35^ ApoB shows greater association with CHD risk than LDL cholesterol or TG levels in a Mendelian randomization model,^36^ although Krauss stressed that ApoB, as a measure of all atherogenic particles, particularly those in LDL, can misrepresent the sdLDL level. He commented on the notion of residual risk beyond LDL cholesterol‐lowering, which may be associated with elevations in the LDL‐like particle lipoprotein(a)^37^ or in other risk factors, but which has led to a recognition that progressively lower LDL cholesterol levels have come to be thought of as optimal targets.

E Dale Abel, Iowa City, IA, discussed relationships between heart failure (HF) and insulin resistance. Diabetes is associated with doubling of rates of HF, with likelihood greater in T1D than T2D,^38^ so that nearly half of persons with HF have diabetes, particularly with preserved ejection fraction.^39^ Furthermore, there is evidence of subclinical myocardial damage in persons with T2D not having clinical CVD,^40^ and the pathogenesis of HF appears to resemble that of insulin resistance itself, including elevation in FFA and proinflammatory cytokine levels, ectopic lipid accumulation, and hyperglycemia itself,^41^ with a meta‐analysis of 10 studies of 178 929 persons with diabetes and 14 176 HF cases showing a 15% increase in likelihood of HF for each 1% increase in HbA1c.^42^ Myocardial glucose toxicity appears to have a variety of potential effects on nuclear and mitochondrial function,^43^ with further effects of lipotoxicity, and reactive oxidative stress impairing mitochondrial function along with the effect of insulin resistance and obesity leading to abnormal contractility.^44^

Gerald Shulman, New Haven, CT, discussed insulin action and insulin resistance in the kidney, noting that the kidney is a gluconeogenic organ. Shulman suggested a teleologic explanation that insulin resistance acts as a defense against starvation.^45^ In the kidney, starvation increases lipolysis leading to accumulation of protein kinase C‐epsilon in renal cortical cell membranes, resulting in insulin resistance.^46^, ^47^

Ronald Evans, La Jolla, CA, discussed the suppression of adipocyte lipolysis by convergent pathways under the influence of insulin and fibroblast growth factor (FGF)1, FGF1 activating phosphodiesterase 4D (PDE4D), whereas insulin inhibits PDE3B.^48^ The physiologic role of FGF1 is as a paracrine/autocrine mediator, the “immediate product” of PPARγ, leading to the concept that “the adipocyte is the control center for insulin resistance,” Evans described the potential that FGF1 can be “endocrinzed” to act as an insulin sensitizer.^49^ He noted that FGF21, produced by skeletal muscle, has similar effects to those of FGF1 in the adipocyte, both promoting lipolysis and acting through the FGF receptor. Both are being studied as potential insulin sensitizing therapeutic agents.
